# Comparing national device-based physical activity surveillance systems: a systematic review

**DOI:** 10.1186/s12966-024-01612-8

**Published:** 2024-07-03

**Authors:** Inge de Wolf, Anne Elevelt, Femke van Nassau, Vera Toepoel, Ellen de Hollander, Maaike E. Kompier, Annemieke Luiten, Barry Schouten, G. C. Wanda Wendel-Vos, Hidde P. van der Ploeg

**Affiliations:** 1 Department of Public and Occupational Health, Amsterdam UMC, Vrije Universiteit Amsterdam, Amsterdam Public Health Research Institute, van der Boechorststraat 7, Amsterdam, 1081BT the Netherlands; 2 Statistics Netherlands, CBS-weg 11, Heerlen, 6412EX the Netherlands; 3 Statistics Netherlands, Henri Faasdreef 312, 2492JP, The Hague, the Netherlands; 4 National Institute for Public Health and the Environment, Antonie van Leeuwenhoeklaan 9, 3721MA, Bilthoven, the Netherlands

**Keywords:** Accelerometer, Pedometer, Physical activity, Sedentary behaviour, Surveillance

## Abstract

**Background:**

Physical activity surveillance systems are important for public health monitoring but rely mostly on self-report measurement of physical activity. Integration of device-based measurements in such systems can improve population estimates, however this is still relatively uncommon in existing surveillance systems. This systematic review aims to create an overview of the methodology used in existing device-based national PA surveillance systems.

**Methods:**

Four literature databases (PubMed, Embase.com, SPORTDiscus and Web of Science) were searched, supplemented with backward tracking. Articles were included if they reported on population-based (inter)national surveillance systems measuring PA, sedentary time and/or adherence to PA guidelines. When available and in English, the methodological reports of the identified surveillance studies were also included for data extraction.

**Results:**

This systematic literature search followed the PRISMA guidelines and yielded 34 articles and an additional 18 methodological reports, reporting on 28 studies, which in turn reported on one or multiple waves of 15 different national and 1 international surveillance system. The included studies showed substantial variation between (waves of) systems in number of participants, response rates, population representativeness and recruitment. In contrast, the methods were similar on data reduction definitions (e.g. minimal number of valid days, non-wear time and necessary wear time for a valid day).

**Conclusions:**

The results of this review indicate that few countries use device-based PA measurement in their surveillance system. The employed methodology is diverse, which hampers comparability between countries and calls for more standardized methods as well as standardized reporting on these methods. The results from this review can help inform the integration of device-based PA measurement in (inter)national surveillance systems.

**Supplementary Information:**

The online version contains supplementary material available at 10.1186/s12966-024-01612-8.

## Background

The importance of physical activity (PA) for our health is well-known [1]. In 2016, it was estimated that 27.5% of the global adult population was insufficiently physically active [2]. Estimates suggest that physical inactivity is responsible for 10% of deaths worldwide [3] and increases the risk for developing chronic diseases such as cardiovascular disease, diabetes and several forms of cancer [4]. The WHO (World Health Organization) 2020 guidelines [5] on PA and sedentary behaviour (SB) recommend adults to undertake 150–300 min of moderate-to-vigorous intensity PA (MVPA) per week and to do muscle-strengthening activities at least two days a week. Furthermore, daily SB should be limited.

To assess and report on these PA and SB levels and guidelines adherence, national population-based surveillance systems are in place to support public health policies and strategies [6]. Since 2004 the WHO recommends to systematically monitor PA in national surveillance systems [7]. Between 2001 and 2016, population-based surveillance has been done in 168 countries worldwide [2]. National surveillance systems differ in what aspects they assess. Both PA, SB and sport participation are measured in different ways and the degree of inquiry of these three varies widely [8].

There are a variety of different methods to assess PA and SB: such as questionnaires, pedometers, accelerometers and heart rate monitoring [9]. The most common method to monitor PA (and SB) in national surveillance systems is by sending out questionnaires [8, 10]. Questionnaires can offer comprehensive information on various dimensions, including domain, type, intensity, frequency, duration and context [11, 12]. However, questionnaires are also prone to social desirability, recall bias and over reporting [11–14]. In addition, the different questionnaires to assess PA and SB vary on a variety of variables: period of interest (e.g., past week, past month, regular week), categories of activity that are included (e.g., leisure, occupation, transport), input (e.g., duration, frequency, intensity) and output (e.g., hours, minutes, total energy expenditure) [15]. As a result, the percentage of participants meeting the PA recommendations can vary between 26% and 92% depending on the questionnaire and guideline that is used [16]. Geographical and cultural differences are currently also an issue with questionnaires when comparing estimates of PA and SB levels across countries [17]. The methodological variations and the resulting variation in reported outcomes make it difficult to give a reliable estimate of PA levels of populations based on questionnaires [16].

Next to that, correlations of PA questionnaires with device-based measures are generally poor to modest [12, 17]. Questionnaires often tend to overestimate PA for both amount of PA and intensity levels when compared to objective measures [18, 19]. Hence, adherence to PA guidelines derived from self-report should be interpreted cautiously [17].

Although questionnaires are the most common method to assess PA, technological advances have led to the growing popularity of device-based PA measurement methods, such as accelerometers. Wearable, accelerometer-based activity monitors have become more commonly used in relatively large national samples to measure PA and SB levels [20–22]. Accelerometer-based activity monitors can measure frequency, intensity and duration of activities, and give information on activity bouts (periods of continuous PA) [23, 24]. These device-based PA measurements have shown to be more reliable and valid compared to questionnaires for assessing PA levels over time [19, 25]. Therefore, the use of activity monitors to assess PA and SB might help to overcome limitations associated with self-reported PA. Implementation of device-based measurement methods in surveillance systems is therefore recommended [17].

When conducting PA research with device-based measurements on a population level numerous methodological decisions have to be made. This includes the type of device, number of wear days, attachment place but also sample selection, data collection and data processing aspects [22, 26]. Furthermore, algorithms are used to separate wake, sleep and non-wear times and estimate intensity levels [24, 27]. With this review, we aim to create an overview of the methodology used in existing device-based PA surveillance systems, which can help inform the development of such systems in other countries and help harmonise data collection in the future.

## Methods

In order to produce such a systematic overview, we conducted a systematic literature review. In preparation of the literature search a review protocol was written, specifying inclusion and exclusion criteria for the studies and data analysis. We registered this review (CRD42022329755) in the PROSPERO database [28]. The reporting of this systematic review adheres to the reporting items of the PRISMA 2020 checklist [29].

### Search strategy

To identify all relevant publications we conducted systematic searches in the bibliographic databases of PubMed, Embase.com, SPORTDiscus (Ebsco) and Web of Science (Core Collection) from inception to March 15th 2024, in collaboration with a medical information specialist at the University Library. The following terms were used (including synonyms and closely related words) as index terms or free-text words: “Accelerometry”, “Actigraphy”, “Actimetry”, “PA”, “Physical inactivity”, “Population surveillance”.

Duplicate papers were removed. The full search strategies for all databases can be found in the Supplementary Material (Additional file 1). In addition, after the full-text review phase, the reference lists of the included papers were also scanned for relevant publications. When references to methodological reports or -papers of the studied surveillance system were given, these were also recovered in order to gain the most complete insight in the used methods of that system.

### Article selection

Only peer-reviewed, English journal papers, dissertations and reports of relevant surveillance systems were included. Laboratory, intervention, cohort and methodological studies were excluded. The papers described studies using national population-based samples of people aged > 16 years that used device-based measurements to measure PA. Papers on studies done in a particular setting (e.g., hospital, school) were excluded. To be included in the study the papers had to report on PA or SB variables, for example, minutes of MVPA, sedentary time, or adherence to PA guidelines.

All retrieved papers were imported into reference manager software (Rayyan and EndNote). Two researchers (IdW, AE) were involved in the article selection, data extraction and quality assessment. For title and abstract selection the two researcher first checked agreement by screening a random sample of 1% of the results together. This was followed by a random sample of 200 that was screened by both researchers, but independently. The remaining results were divided and screened independently. In case of uncertainty, the paper was included for full text review. For the full-text selection and quality assessment both researchers screened all full text papers independently. The few differences in judgement were resolved through a consensus meeting between the two reviewers.

### Data extraction

A standardized, pre-piloted form was used to extract data from the included studies, this was done by one researcher (IdW). In case of uncertainty it was discussed with another researcher. The extracted data included study characteristics, population characteristics, data collection methods, data processing, data analyses and outcome measures.

**Fig. 1:**
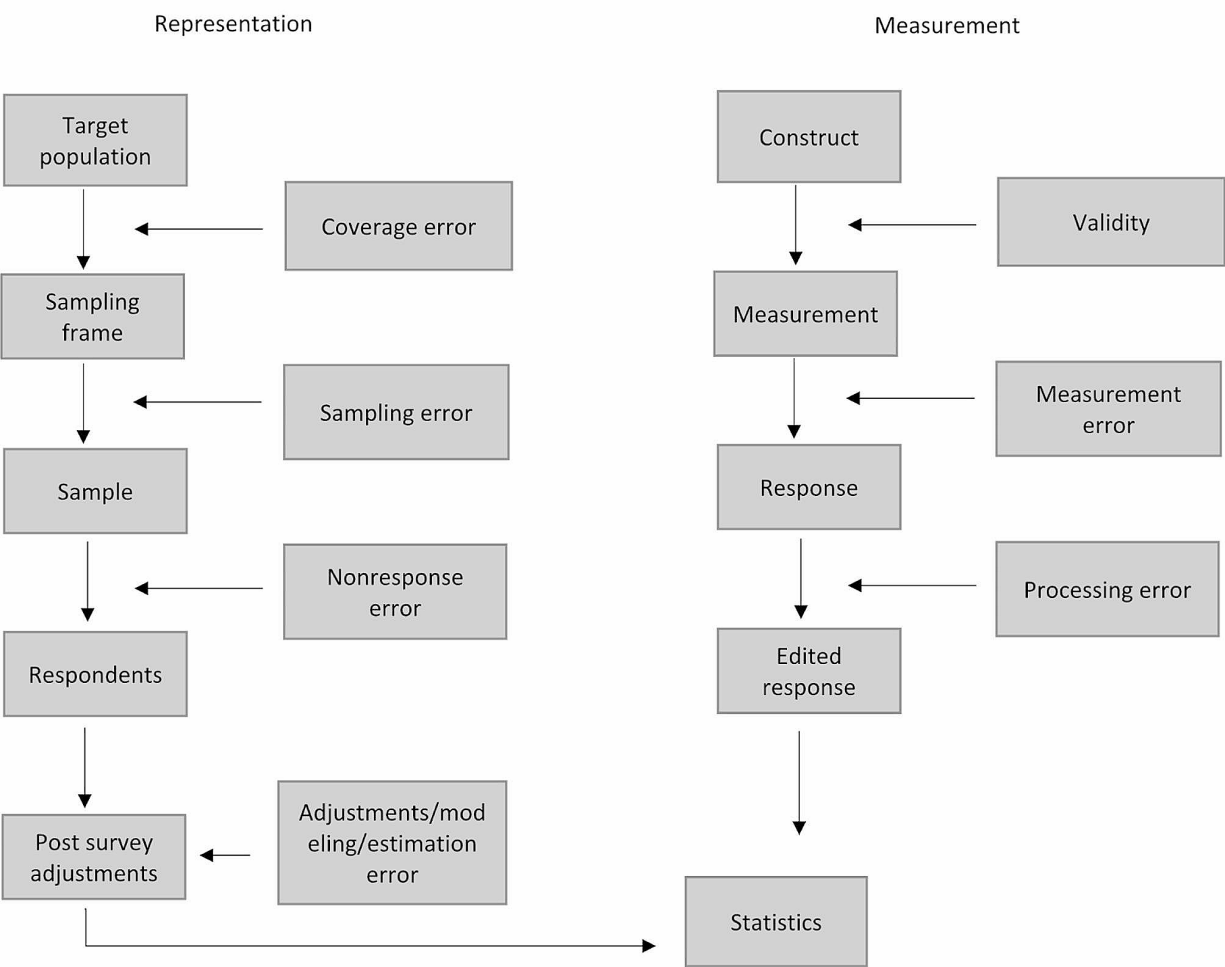
Components of the Total Survey Error Framework

The quality of the included surveillance systems was linked to the Total Survey Error Framework (TSEF) [30]. This framework can be seen in Fig. 1. This framework consists of two dimensions in which possible sources of error can occur. Within these dimensions the framework describes a set of quality concepts. The first four sources of errors (coverage-, sampling, nonresponse- and adjustment error) are the quality concepts about the representativeness of the study and start with the target population and end at the post survey adjustments. The other sources of error are related to measurement and consist of three quality concepts (validity, measurement- and processing error). The concepts of this framework were linked to PA surveillance systems to identify specific areas of potential improvement in reporting and methodology.

## Results

### Study selection

**Fig. 2:**
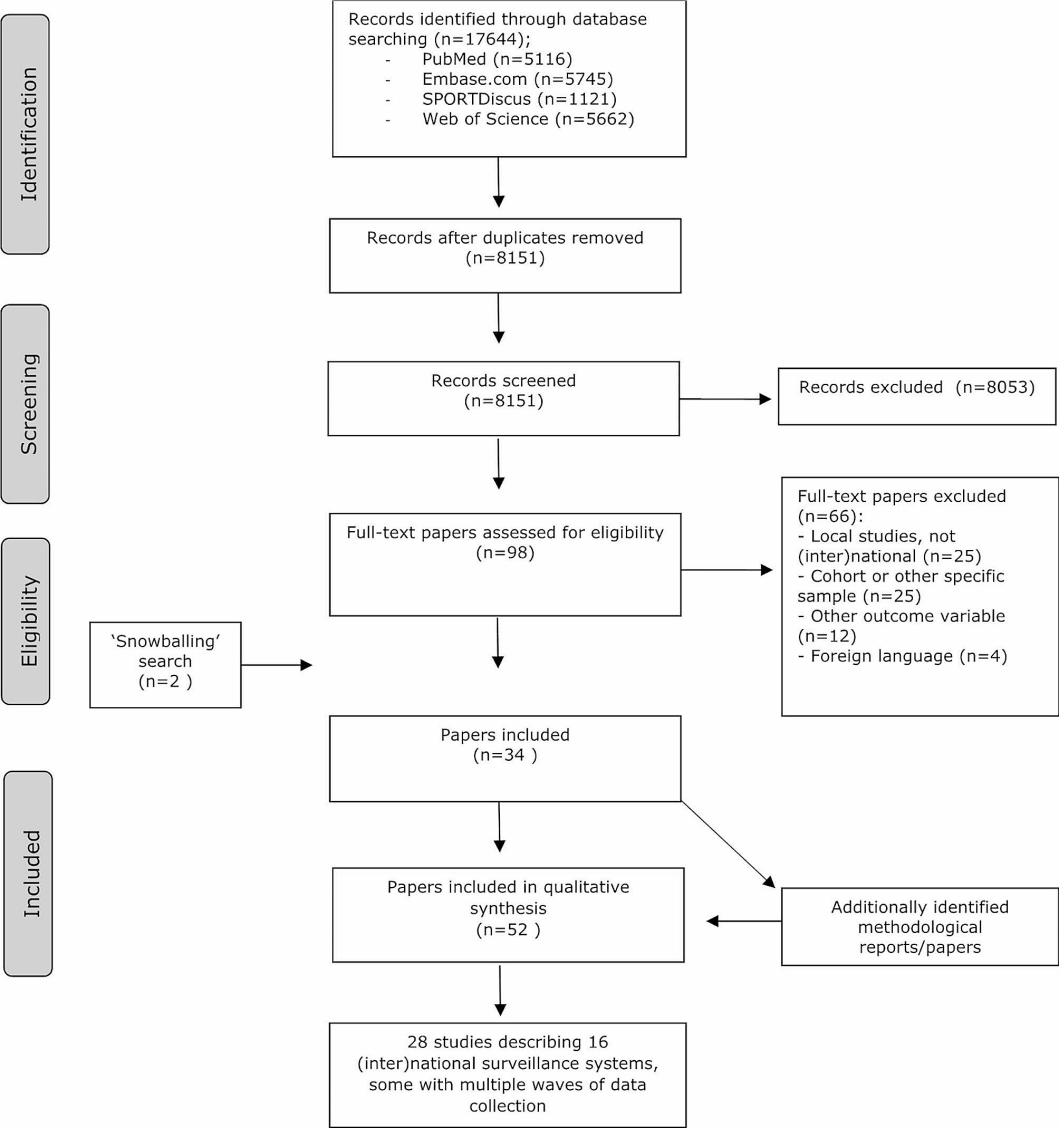
Flowchart of the search and selection procedure of studies

The flow chart of the search and selection process is presented in Fig. 2. After screening 8151 titles and abstracts, 98 full-text papers were assessed for eligibility. Sixty-six papers were excluded based on the exclusion criteria, leaving 32 papers for final inclusion. Most papers were excluded because they assessed PA in local cohorts. Two additional papers were found in the reference lists of the eligible papers resulting in a total of 34 included papers [31–64]. The 34 included papers described 28 studies that used device-based measurement to monitor PA and or SB on an (inter)national level, covering 15 different national surveillance systems and 1 international surveillance system. In some papers one or more methodological reports/papers on the studied surveillance system were referenced. We identified and included 18 additional methodological reports/papers [65–82]. Finally, we obtained additional information through correspondence with the authors of the included studies by email.

### Study information and sample characteristics

The study information and sample characteristics of the included papers are shown the Supplementary Material (Additional file 2 Tables 1 and 2). Surveillance systems that use accelerometers were separated from the systems using pedometers. This was done because not all aspects from the data extraction are applicable to both.

Most of the surveillance systems were from European countries (*n* = 8) [31–33, 38, 42–53, 56–59]and North-America (*n* = 3) [34–37, 39–41, 50, 60–64]. The other systems were from Asia (*n* = 3) [38, 54, 59] and South-America (*n* = 2) [43–45]. Only two systems were from low- and middle-income countries (LMIC) [43–45]. The oldest surveillance system originated from 1995 (Japan) [54], while the most recent identified paper is on the Finnish second wave from 2017/19 [39, 53]. Half of the systems used device-based measurement during one wave only (*n* = 7) [31, 32, 38, 42–45, 50, 57, 59]. The other half of the systems (Finland, Norway, Sweden, Canada, Denmark, Portugal, United States and Japan) [33–37, 39–41, 46–49, 51–58, 60–64] collected more than one wave of surveillance with device-based measurements. In seven systems device-based measurement was only applied in a sub-sample [31, 32, 38, 43–45, 50–52, 56, 59]. The number of participants ranged from 224 for the first wave in Denmark in 2007 [56] to more than 6000 participants in a single wave for Japan, Portugal and the United States [33, 54, 55, 58, 60, 62–64].

Response rates ranged from 17.6% in Finland for a completely new sample when calculating it from the total potential participants belonging to the sample [53], to 98% in Brazil for a sub-sample when participants were selected from the participants of an original study [45]. Besides, response rates were calculated in different ways (i.e., response rates were sometimes reported as people that complied with the wear time protocol, or calculated from different original samples) and some studies did not report response rates at all or lacked sufficient information to calculate them [34–37, 39–41, 43, 44, 47, 55, 59–64]. Almost all studies reported having nationally representative samples and having selected participants with a variety of probability sampling methods. Nonresponse analyses were not always performed or reported. When this was done it was most often for gender and age [31, 32, 43, 44, 46–48, 50] and less frequent for educational level and body mass index (BMI) [42, 45, 51–53]. Differences between respondents and non-respondents were not frequently reported. The adjustments for nonresponse bias and other biases were also not always performed or reported. The first two quality concepts of the TSEF (coverage- and sampling error) are mostly well described and executed in the identified surveillance systems. More room for improvement can be found for the other two quality concepts (nonresponse error and adjustment error). Recruitment was most often done via introductory letters followed by a call or visit from an interviewer. Canada and Norway used media coverage in the first waves [41, 48] to facilitate recruitment and two studies recruited from schools, work sites and community settings [33, 55, 58].

### Device information and data collection methods

Tables 3 and 4 in the Supplementary Material (Additional file 2 Tables 3 and 4) give an overview of device information and data collection methods of the included papers. A variety of different monitors were used in the surveillance systems. The most used devices are different models of the ActiGraph accelerometer. Finland used the Hookie in wave 1 [51, 52] and the UKKRM42 in wave 2 [53], while Barbados [50] used the Actiheart and Canada the Actical in all their five waves [37, 39–41]. All countries using a pedometer (Czech republic, Denmark and Japan) used a Yamax [54, 56, 57]. The monitors were worn on the right hip/waist/lower back, except for Barbados were participants had to wear the device on the chest [50], and for the third and fourth wave in the United States and in Luxembourg where it was worn on the wrist [35, 42, 61]. For the majority of the studies, participants were instructed to wear the monitor for all waking hours and only to take it off during water-based activities [31–34, 36–41, 43–49, 51, 52, 55, 58–60, 62–64]. In Barbados [50], the second wave in Finland [53], the third and fourth wave in the United States [35, 61] and in Luxembourg [42] participants were asked to wear the monitor continuously, so also when sleeping. In Finland, participants were asked to wear the device on the right hip during waking hours and on the non-dominant wrist when sleeping. In most surveillance systems the distribution of the device was done in person (*n* = 10) [31–33, 36–45, 51–53, 58–60, 62–64]. Only Norway [48] and Sweden [47] specified for wave 1 that they distributed the accelerometers via mail. Epoch length used for analyses was usually set at 60 s but ranged from 10 [48] to 60 s. The monitoring period ranged from one to seven days, with Japan [54] measuring during one “typical” day, Barbados [50] during four days and for all other systems during seven consecutive days. Wear-time compliance ranged from 59% in England [31, 32] to 97% in Barbados [50] and was most often around 80–90%. For some systems wear time compliance was not reported (waves 2 and 3 Canada [39, 40], wave 2 Sweden [46], Portugal [55], and Japan [54]). Regarding financial incentives, only England [31, 32] and the United States [34–36, 60–64] reported giving a £20,- gift voucher and $40 respectively. Some systems gave feedback to the participants on their own PA levels as an incentive [33, 48, 49, 51–53, 58].

### Data processing and outcome measures

Information on data processing and outcome measures for the included studies are shown in Tables 5 and 6 of the Supplementary Material (Additional file 2 Tables 5 and 6). In the majority of studies four days with valid data were required, with the exception of four systems: for the second wave in Norway [49] and Japan [54] one day with valid data was sufficient, while Brazil [45] and the Latin America study [43, 44] required at least five days, including one weekend day. Regarding required wear-time, a valid day was defined by almost all studies as at least 10 h of wear-time per day. For Barbados and the second wave in Finland a wear time of 24 h/d was required [41, 51, 72], for the third and fourth wave of the United states 23 h/d was required [35, 61]. For non-wear time various definitions were used: the most common definition for non-wear time was any period of continuous zero counts for 60 or more consecutive minutes. In the second wave in Finland [53] 120 min of continuous zero counts was considered non-wear time, while for the first wave in Sweden [47] this was set at 20 min.

PA intensities were estimated based on different cut-points linking activity counts to metabolic equivalents (METs). For the ActiGraph the cut-points from Troiano [62] and Freedson (113) were most often used. Studies that reported on outliers used a variety of cleaning rules. The most common outcome measures were adherence to the PA guidelines and minutes per day at different PA intensities. Meeting the PA guidelines in most studies meant accumulating at least 150 min of MVPA per week in bouts of 10 min or more. For England and Luxembourg the bouts of at least 10 min were not required [31, 32, 42] and Norway did not specify their used PA guidelines [48, 49]. For systems using pedometers, steps per day as a continuous variable was the most commonly reported outcome.

## Discussion

In this systematic review we aimed to map the methodology used in existing national PA surveillance systems using device-based measurement. This was done to support the development of such systems in other countries and to harmonise data collection in the future. A total of 34 eligible papers were identified, reporting on fifteen national and one international surveillance system. Substantial variation between waves of systems was found in number of participants, response rates, population representativeness and recruitment. Data reduction definitions were more often comparable between systems (e.g., minimal number of valid days, non-wear time and necessary wear time for a valid day). This was not the case within the systems using pedometers. Pedometers were the forerunners of accelerometers, but are now less often used for population surveillance [83], and more as behavioral chance tools in interventions. For this reason, we focus more on the systems using accelerometers in the discussion. Because data was collected before the launch of the 2020 WHO guidelines on PA and SB, they refer to the WHO 2010 guidelines that required MVPA to be accumulated in bouts of at least 10 min. In the 2020 guidelines all MVPA counts, regardless of the duration of the bouts in which it was accumulated [5]. Only the paper using the data from Luxembourg refers to both WHO guidelines [42].

### Representativeness in PA surveillance with device-based measurement

A national surveillance system aims to assess the PA levels of the whole population. Representativeness of the study sample is therefore an important aspect to consider when conducting PA surveillance with device-based measurement. Most systems identified in our review claim to have “a nationally representative sample” or “nationally representative data”. However, response rates in the included studies were as low as 17.6% and regularly around 50%. In many PA intervention trials participants tend to be women, Caucasian and higher educated compared to nonparticipants [84]. The people that participated in the included studies might therefore differ from the whole original sample, resulting in potential nonresponse bias. It is difficult to determine if the participating sample is truly representative of characteristics of the whole population. Using for example census data, it would be possible to weigh the participating sample towards the population distributions for factors such as age, gender, income and area of residence. However, representativeness of other relevant factors, such as health status, is not always possible. Another issue is the representativeness of samples with regard to PA itself. Population PA levels are often not known (which is why the surveillance system is in place) so there is no data to calibrate the surveillance system sample against. Hence, it is not possible to determine if, for example, more active people are more likely to participate in the surveillance system which would result in an overestimation of population PA levels. Often the only way to attempt to adjust for such potential confounding is by weighing the population towards the known population distribution for the factors mentioned above (e.g., age, gender, income). However, it remains to be seen if selection bias towards PA level for example can be adjusted for in this way and hence if the participating sample is truly representative of the population PA levels.

Not all identified surveillance systems performed nonresponse analyses and the surveillance systems that did a non-response bias analysis only looked at basic demographic variables such as gender, age, education level and sometimes body mass index. To avoid biased PA estimates in survey research it is preferable to adjust for non-response error [85]. We believe that non-response bias analysis should also be done on PA levels. This was not done in the included studies in this review. As PA data are usually unavailable for non-respondents it is hard to know if the weights and other adjustment procedures in the surveillance systems are sufficient to keep the results representative of the whole population.

Non-response bias and the related adjustment procedures are important to consider when conducting PA surveillance with devices. Claims of population representativeness of PA levels need to be substantiated and potential limitations in such representativeness should be discussed.

### Measurement in PA surveillance with device-based measurement

Besides the issues around representativeness mentioned in the previous paragraph, we also want to discuss measurement error and processing error of the measurement side of the TSEF, that show room for improvement.

The placement of the device has implications for the PA outcome measures. Accelerometers cannot detect all types of movements depending on the attachment place. The most common accelerometer placement used in the systems included in this review was worn on the right hip/waist. Accelerometers placed on the hip or waist can underestimate certain movements (e.g., upper body movements, cycling) and can be uncomfortable to wear during sleep [86]. The USA (NHANES) changed the attachment location of the accelerometer from the hip to the wrist after two waves to improve compliance and collect sleep measures [87]. In the second wave in Finland, they used a 24 h approach by changing the accelerometer from the hip during the day to the wrist during the night. This two wear-site approach did not impact compliance and it was found feasible among working-aged adults [53]. Determining the best possible attachment place can be a challenge. Thigh-worn devices might be more suitable to wear 24 h a day as they do not need to change position and lower participant burden [88]. Thigh-worn monitors might therefore be more accurate in predicting time spent in different PA intensity categories and SB breaks compared to hip- and wrist-worn accelerometers [89].

The next quality aspect is the processing error which is connected to the measurement as well. The identified studies used different criteria for valid wear time, which has major implications on the outcome measures of PA. Studies have shown that the amount of wear time influences the amount of PA measured by the device during the day [90, 91]. It also influences the number of individuals having enough valid days to be included [92]. These possible processing errors can influence the survey statistics. Standardization of the data processing would help increase comparability of the results.

### Strengths and limitations

This study provides an overview of all PA surveillance systems worldwide that use device-based measurements. The main strength of this study is the thorough and systematic review process. The search was performed in four databases complimented with additional search strategies (i.e., looking at the references of included papers, looking at the websites of the systems and contacting the authors). In addition, the article selection and quality assessment were conducted independently by two researchers.

Even though the search was extensive the possibility exists that papers or background reports are missed because we only looked for English results. Especially methodological reports and other background reports might have been written in other languages and therefore be missed by the search strategy.

Although some methodological reports might have been missed because of the language restrictions, a strength of this study is the inclusion of those background reports, which have provided extra information. Several of the 34 papers that were eligible for inclusion reported on the same study. We decided to combine all identified papers and reports to describe the methodology used for that particular system in the best possible way.

A limitation of this study is the lack of a quality assessment using a quality assessment tool. Currently, to our knowledge, no quality assessment tool for surveillance systems exists. To overcome this we used the TSEF [30] as an alternative to look at the quality and identify aspects with room for improvement.

The identified papers presented on data collected from 1995 to 2019. More recent (ongoing) studies might not have been found in the search as it can take time before the data becomes available. A check of the websites of the included surveillance systems revealed more recent or ongoing waves on which no publications were available yet. A new wave of CHMS (Canada, 2019) and FinFit (Finland, 2022) have recently been finished. CHMS 8 (Canada) and Kan 3 (Norway) are ongoing at the time of writing this paper. These cycles have not been included in this paper since results were not yet presented at the time of writing this paper.

Another strength of this review is the extensive data extraction. This gives an overview of the aspects that the included surveillance systems have described, but also which methodological aspects have limitations. However, with limited available space in papers it might be hard to report on all aspects. Our paper might therefore not describe the quality aspects of the surveillance system but mainly the quality of reporting in the identified papers and reports.

### Recommendations

The results of this review highlight the need for standardisation of measurement methods used in device-based surveillance systems worldwide. The different methodological aspects reported and the incompleteness of information on certain system aspects made it difficult to compare systems and create a complete overview. More standardized reporting would enable better comparison of the surveillance systems and create a complete overview of all important aspects to consider when implementing device-based PA monitoring.

For surveillance systems representativeness is especially important. According to the used quality framework [30] both nonresponse and adjustment error are important quality aspects to consider. Information to assess these aspects was not always reported or investigated. We advocate for more standardized reporting on these aspects. Standardized reporting on the difference between respondents and non-respondents on as many variables as possible should be the goal. To be able to do this, clear reporting on the sample, number of participants and the associated response rates are necessary. If necessary, the adjustments in the face of, for example, nonresponse error to improve the sample estimate should be reported. The framework might be a helpful tool to improve reporting on these steps in a more standardized manner.

Additionally, the framework might also be helpful to further standardize the measurement dimension [30]. Considering the validity, measurement error and processing error can result in more standardized methods. Even though there is some standardization in certain methodological aspects, there is generally insufficient standardization to compare different national device-based PA surveillance systems [25].

Furthermore, this review showed that 15 countries currently employ a device-based surveillance system which may be implemented by more countries. Using device-based measurement in PA surveillance systems can provide more accurate and comparable estimates of PA and SB levels across countries and over time [17, 19, 25]. An important issue here is the affordability as device-based data are more expensive to collect than questionnaire data. Therefore, device-based measurements are harder to implement in surveillance systems, especially in LMIC. Nonetheless, two surveillance systems in South American LMIC have already used device-based measurements [43–45]. Furthermore, the WHO has also piloted wrist-worn accelerometery in Africa (Malawi) to determine its feasibility for implementation into the global WHO STEPS surveillance system (STEPwise approach to noncommunicable disease risk factor surveillance) [93]. They concluded that it is feasible to implement devices within the STEPS surveillance system in countries such as Malawi. Hence, the standardisation of methodologies across countries that STEPS facilities could work for device-based methods in addition to the more commonly used questionnaire based methods that are likely to still employed in many countries, at least in the short term.

Ideally a combination of device-based measurements and questionnaires in surveillance systems should be implemented. The differences in the dimensions studied by questionnaires and devices make these two methods complementary to each other. Combining both methods and fine tuning their complementary outcomes in a surveillance system will result in the most complete picture of national physical activity levels [25]. Device-based measurements will provide more valid data about intensity and duration while questionnaires are useful for information about context and activity types and domains [17, 94]. However, more research on how to best utilize device-based data in synergy with questionnaire data for national surveillance would be helpful.

## Conclusion

In this systematic review, we mapped the methodology used in existing national PA surveillance systems using device-based measurement. Currently, only fifteen countries have monitored population level PA with device-based measurements. Although they do not vary much in the device used, differences are found in many other methodological aspects and harmonization of methodologies would improve comparability. Representativeness of surveillance systems with regard to PA levels is an important aspect that needs attention in the future. This review and the associated recommendations can be used to incorporate device-based measurement of PA in surveillance systems of other countries.

### Electronic supplementary material

Below is the link to the electronic supplementary material.

Supplementary Material 1

Supplementary Material 2

## Data Availability

All data generated or analysed during this study are included in this published article and its supplementary information files.

## Abbreviations

PA: Physical activity
WHO: World Health Organization
SB: Sedentary behaviour
MVPA: Moderate-to-vigorous intensity physical activity
TSEF: Total Survey Error Framework
BMI: Body mass index
LMIC: Low- and middle-income countries
MET: Metabolic equivalent of task
