# Supplementary material for: Comparing national device-based physical activity surveillance systems: a systematic review
Source: Int J Behav Nutr Phys Act. 2024 Jul 3;21:67. doi: 10.1186/s12966-024-01612-8 (PMC11223351; doi:10.1186/s12966-024-01612-8)
Supplement: Supplementary file 1 — Supplementary Material 1 [file 12966_2024_1612_MOESM1_ESM.docx]

**Additional file 1.** Full search strategies

**PubMed Session Results (15 Mar 2024)**

| Search | Query | Items found |
| --- | --- | --- |
| #4 | **#1 AND #2 AND #3** | 5,116 |
| #3 | **"Population Surveillance"[Mesh] OR "national*"[tiab] OR "international*"[tiab] OR "multinational*"[tiab] OR "nationwide"[tiab] OR "population*"[tiab] OR "representative sample*"[tiab]** | 3,239,235 |
| #2 | **"activity monitor*"[tiab] OR (("measur*"[tiab] OR "monitor*"[tiab] OR "evaluat*"[tiab] OR "determin*"[tiab] OR "describ*"[tiab]) AND ("Motor Activity"[Mesh:NoExp] OR "Exercise"[Mesh] OR "Sports"[Mesh] OR "Physical Exertion"[Mesh] OR "motor activit*"[tiab] OR "physical activit*"[tiab] OR "physically activ*"[tiab] OR "active lifestyle*"[tiab] OR "active behav*"[tiab] OR "locomotor activit*"[tiab] OR "activity level*"[tiab] OR "level of activit*"[tiab] OR "exercis*"[tiab] OR "motion"[tiab] OR "Sedentary behavior"[Mesh] OR "Metabolic Equivalent"[Mesh] OR "Screen Time"[Mesh] OR "metabolic equivalent*"[tiab] OR "sedent*"[tiab] OR "inactive lifestyle*"[tiab] OR "inactive behav*"[tiab] OR "inactivity level*"[tiab] OR "physical inactivit*"[tiab] OR "computer time"[tiab] OR "TV time"[tiab] OR "screen time"[tiab] OR "stationary behavio*"[tiab] OR "sitting"[tiab]))** | 586,139 |
| #1 | **"Accelerometry"[Mesh] OR "acceleromet*"[tiab] OR "actigraph*"[tiab] OR "ActivPAL*"[tiab] OR "UKK"[tiab] OR "Actiheart"[tiab] OR "Actical"[tiab] OR "GeneActiv"[tiab] OR "RT3"[tiab] OR "actimet*"[tiab] OR "activity track*"[tiab] OR "fitness track*"[tiab] OR "Wearable Electronic Devices"[Mesh:NoExp] OR "Fitness Trackers"[Mesh] OR "sensor-assist*"[tiab] OR "motion-sensor*"[tiab] OR "sensorassist*"[tiab] OR "motionsensor*"[tiab] OR (("wearable"[tiab] OR "wearables"[tiab] OR "bodyworn*"[tiab] OR "body-worn*"[tiab] OR "wristworn*"[tiab] OR "wrist-worn*"[tiab] OR "wristbased"[tiab] OR "wrist-based"[tiab] OR "thigh-worn*"[tiab] OR "thighworn*"[tiab] OR "hip-worn*"[tiab] OR "hipworn*"[tiab] OR "thigh-based*"[tiab] OR "thighbased*"[tiab] OR "hip-based*"[tiab] OR "hipbased*"[tiab]) AND ("device*"[tiab] OR "technolog*"[tiab] OR "sensor*"[tiab] OR "monitor*"[tiab]))** | 65,350 |

**Embase.com Session Results (15 Mar 2024)**

| Search | Query | Items found |
| --- | --- | --- |
| #4 | **(#1 AND #2 AND #3) NOT ('conference abstract'/it OR 'conference review'/it)** | 5,745 |
| #3 | **'epidemiological surveillance'/exp OR** **'national*':ab,ti,kw OR 'international*':ab,ti,kw OR 'multinational*':ab,ti,kw OR 'nationwide':ab,ti,kw OR 'population*':ab,ti,kw OR 'representative sample*':ab,ti,kw** | 4,692,917 |
| #2 | **'activity monitor'/exp OR 'activity monitor*':ab,ti,kw OR (('measur*':ab,ti,kw OR 'monitor*':ab,ti,kw OR 'evaluat*':ab,ti,kw OR 'determin*':ab,ti,kw OR 'describ*':ab,ti,kw) AND ('motor activity'/de OR 'exercise'/exp OR 'sport'/exp OR 'physical activity'/exp OR 'motor activit*':ab,ti,kw OR 'physical activit*':ab,ti,kw OR 'physically activ*':ab,ti,kw OR 'active lifestyle*':ab,ti,kw OR 'active behav*':ab,ti,kw OR 'locomotor activit*':ab,ti,kw OR 'activity level*':ab,ti,kw OR 'level of activit*':ab,ti,kw OR 'exercis*':ab,ti,kw OR 'motion':ab,ti,kw OR 'sedentary lifestyle'/exp OR 'sedentary time'/exp OR 'active lifestyle'/exp OR 'metabolic equivalent'/exp OR 'screen time'/exp OR 'metabolic equivalent*':ab,ti,kw OR 'sedent*':ab,ti,kw OR 'inactive lifestyle*':ab,ti,kw OR 'inactive behav*':ab,ti,kw OR 'inactivity level*':ab,ti,kw OR 'physical inactivit*':ab,ti,kw OR 'computer time':ab,ti,kw OR 'TV time':ab,ti,kw OR 'screen time':ab,ti,kw OR 'stationary behavio*':ab,ti,kw OR 'sitting':ab,ti,kw))** | 898,570 |
| #1 | **'accelerometry'/exp OR 'accelerometer'/exp OR 'actimetry'/exp OR 'actigraph'/exp OR 'activpal'/exp OR 'activpal3'/exp OR 'actiheart'/exp OR 'geneactiv'/exp OR 'acceleromet*':ab,ti,kw OR 'actigraph*':ab,ti,kw OR 'ActivPAL*':ab,ti,kw OR 'UKK':ab,ti,kw OR 'Actiheart':ab,ti,kw OR 'Actical':ab,ti,kw OR 'GeneActiv':ab,ti,kw OR 'RT3':ab,ti,kw OR 'actimet*':ab,ti,kw OR 'activity track*':ab,ti,kw OR 'fitness track*':ab,ti,kw OR 'wearable computer'/de OR 'motion sensor'/exp OR 'wearable sensor'/exp OR 'sensor-assist*':ab,ti,kw OR 'motion-sensor*':ab,ti,kw OR 'sensorassist*':ab,ti,kw OR 'motionsensor*':ab,ti,kw OR (('wearable':ab,ti,kw OR 'wearables':ab,ti,kw OR 'bodyworn*':ab,ti,kw OR 'body-worn*':ab,ti,kw OR 'wristworn*':ab,ti,kw OR 'wrist-worn*':ab,ti,kw OR 'wristbased':ab,ti,kw OR 'wrist-based':ab,ti,kw OR 'thigh-worn*':ab,ti,kw OR 'thighworn*':ab,ti,kw OR 'hip-worn*':ab,ti,kw OR 'hipworn*':ab,ti,kw OR 'thigh-based*':ab,ti,kw OR 'thighbased*':ab,ti,kw OR 'hip-based*':ab,ti,kw OR 'hipbased*':ab,ti,kw) AND ('device*':ab,ti,kw OR 'technolog*':ab,ti,kw OR 'sensor*':ab,ti,kw OR 'monitor*':ab,ti,kw))** | 87,587 |

**SPORTDiscus (Ebsco) Session Results (15 Mar 2024)**

| Search | Query | Items found |
| --- | --- | --- |
| S4 | **S1 AND S2 AND S3** | 1,121 |
| S3 | **DE "HEALTH surveys" OR TI ("national*" OR "international*" OR "multinational*" OR "nationwide" OR "population*" OR "representative sample*") OR AB ("national*" OR "international*" OR "multinational*" OR "nationwide" OR "population*" OR "representative sample*") OR KW ("national*" OR "international*" OR "multinational*" OR "nationwide" OR "population*" OR "representative sample*")** | 332,803 |
| S2 | **DE "MOTION capture (Human mechanics)" OR DE "PHYSICAL activity measurement" OR TI ("activity monitor*" OR (("measur*" OR "monitor*" OR "evaluat*" OR "determin*" OR "describ*") AND ("motor activit*" OR "physical activit*" OR "physically activ*" OR "active lifestyle*" OR "active behav*" OR "locomotor activit*" OR "activity level*" OR "level of activit*" OR "exercis*" OR "motion" OR "metabolic equivalent*" OR "sedent*" OR "inactive lifestyle*" OR "inactive behav*" OR "inactivity level*" OR "physical inactivit*" OR "computer time" OR "TV time" OR "screen time" OR "stationary behavio*" OR "sitting"))) OR AB ("activity monitor*" OR (("measur*" OR "monitor*" OR "evaluat*" OR "determin*" OR "describ*") AND ("motor activit*" OR "physical activit*" OR "physically activ*" OR "active lifestyle*" OR "active behav*" OR "locomotor activit*" OR "activity level*" OR "level of activit*" OR "exercis*" OR "motion" OR "metabolic equivalent*" OR "sedent*" OR "inactive lifestyle*" OR "inactive behav*" OR "inactivity level*" OR "physical inactivit*" OR "computer time" OR "TV time" OR "screen time" OR "stationary behavio*" OR "sitting"))) OR KW ("activity monitor*" OR (("measur*" OR "monitor*" OR "evaluat*" OR "determin*" OR "describ*") AND ("motor activit*" OR "physical activit*" OR "physically activ*" OR "active lifestyle*" OR "active behav*" OR "locomotor activit*" OR "activity level*" OR "level of activit*" OR "exercis*" OR "motion" OR "metabolic equivalent*" OR "sedent*" OR "inactive lifestyle*" OR "inactive behav*" OR "inactivity level*" OR "physical inactivit*" OR "computer time" OR "TV time" OR "screen time" OR "stationary behavio*" OR "sitting"))) OR ((TI ("measur*" OR "monitor*" OR "evaluat*" OR "determin*" OR "describ*") OR AB ("measur*" OR "monitor*" OR "evaluat*" OR "determin*" OR "describ*") OR KW ("measur*" OR "monitor*" OR "evaluat*" OR "determin*" OR "describ*")) AND (DE "EXERCISE" OR DE "SPORTS" OR DE "PHYSICAL activity" OR DE "PHYSICALLY active people" OR DE "SEDENTARY behavior" OR DE "SEDENTARY lifestyles" OR DE "SEDENTARY people" OR DE "METABOLIC equivalent"))** | 120,846 |
| S1 | **DE "ACCELEROMETERS" OR TI ("acceleromet*" OR "actigraph*" OR "ActivPAL*" OR "UKK" OR "Actiheart" OR "Actical" OR "GeneActiv" OR "RT3" OR "actimet*" OR "activity track*" OR "fitness track*" OR "sensor-assist*" OR "motion-sensor*" OR "sensorassist*" OR "motionsensor*" OR (("wearable" OR "wearables" OR "bodyworn*" OR "body-worn*" OR "wristworn*" OR "wrist-worn*" OR "wristbased" OR "wrist-based" OR "thigh-worn*" OR "thighworn*" OR "hip-worn*" OR "hipworn*" OR "thigh-based*" OR "thighbased*" OR "hip-based*" OR "hipbased*") AND ("device*" OR "technolog*" OR "sensor*" OR "monitor*"))) OR AB ("acceleromet*" OR "actigraph*" OR "ActivPAL*" OR "UKK" OR "Actiheart" OR "Actical" OR "GeneActiv" OR "RT3" OR "actimet*" OR "activity track*" OR "fitness track*" OR "sensor-assist*" OR "motion-sensor*" OR "sensorassist*" OR "motionsensor*" OR (("wearable" OR "wearables" OR "bodyworn*" OR "body-worn*" OR "wristworn*" OR "wrist-worn*" OR "wristbased" OR "wrist-based" OR "thigh-worn*" OR "thighworn*" OR "hip-worn*" OR "hipworn*" OR "thigh-based*" OR "thighbased*" OR "hip-based*" OR "hipbased*") AND ("device*" OR "technolog*" OR "sensor*" OR "monitor*"))) OR KW ("acceleromet*" OR "actigraph*" OR "ActivPAL*" OR "UKK" OR "Actiheart" OR "Actical" OR "GeneActiv" OR "RT3" OR "actimet*" OR "activity track*" OR "fitness track*" OR "sensor-assist*" OR "motion-sensor*" OR "sensorassist*" OR "motionsensor*" OR (("wearable" OR "wearables" OR "bodyworn*" OR "body-worn*" OR "wristworn*" OR "wrist-worn*" OR "wristbased" OR "wrist-based" OR "thigh-worn*" OR "thighworn*" OR "hip-worn*" OR "hipworn*" OR "thigh-based*" OR "thighbased*" OR "hip-based*" OR "hipbased*") AND ("device*" OR "technolog*" OR "sensor*" OR "monitor*")))** | 9,935 |

**Web of Science (Core Collection) Session Results (15 Mar 2024)**

| Search | Query | Items found |
| --- | --- | --- |
| #4 | **#1 AND #2 AND #3** | 5,662 |
| #3 | **TS=("national*" OR "international*" OR "multinational*" OR "nationwide" OR "population*" OR "representative sample*")** | 5,351,586 |
| #2 | **TS=("activity monitor*" OR (("measur*" OR "monitor*" OR "evaluat*" OR "determin*" OR "describ*") AND ("motor activit*" OR "physical activit*" OR "physically activ*" OR "active lifestyle*" OR "active behav*" OR "locomotor activit*" OR "activity level*" OR "level of activit*" OR "exercis*" OR "motion" OR "metabolic equivalent*" OR "sedent*" OR "inactive lifestyle*" OR "inactive behav*" OR "inactivity level*" OR "physical inactivit*" OR "computer time" OR "TV time" OR "screen time" OR "stationary behavio*" OR "sitting")))** | 799,354 |
| #1 | **TS=("acceleromet*" OR "actigraph*" OR "ActivPAL*" OR "UKK" OR "Actiheart" OR "Actical" OR "GeneActiv" OR "RT3" OR "actimet*" OR "activity track*" OR "fitness track*" OR "sensor-assist*" OR "motion-sensor*" OR "sensorassist*" OR "motionsensor*" OR (("wearable" OR "wearables" OR "bodyworn*" OR "body-worn*" OR "wristworn*" OR "wrist-worn*" OR "wristbased" OR "wrist-based" OR "thigh-worn*" OR "thighworn*" OR "hip-worn*" OR "hipworn*" OR "thigh-based*" OR "thighbased*" OR "hip-based*" OR "hipbased*") AND ("device*" OR "technolog*" OR "sensor*" OR "monitor*")))** | 106,404 |
