# Supplementary material for: Comparing national device-based physical activity surveillance systems: a systematic review
Source: Int J Behav Nutr Phys Act. 2024 Jul 3;21:67. doi: 10.1186/s12966-024-01612-8 (PMC11223351; doi:10.1186/s12966-024-01612-8)
Supplement: Supplementary file 2 — Supplementary Material 2 [file 12966_2024_1612_MOESM2_ESM.docx]

**Additional file 2 – Tables 1-6**

**Table 1** Study information and sample characteristics of the surveillance systems using accelerometers by country and wave

|  | **Country** | **Surveillance system** | **Article(s), type of source** | **Year(s) of monitoring** | **Sample frame & sampling method** | **Sample size**  **(sub-sample)** | **Number of participants** | **Response rates** | **Non-response analyses** | **Adjustments for biases** | **Age range (mean age) – years** | **Recruitment** |
| --- | --- | --- | --- | --- | --- | --- | --- | --- | --- | --- | --- | --- |
| 1 | Barbados | Sub-study NM | Scientific article (39)  Methodological article (67) | 2011-2013 | Selected from a national sample frame with a probability design | 527 | 364 | 69% | Nonresponse bias analysis for the whole sample compared to the Barbados population have been done for age and gender. Nonresponse bias analysis has been done for demographic, socioeconomic and anthropometric characteristics to compare the PA sub-sample with the whole study sample. | Discrepancies in the whole sample were addressed by the survey weighting scheme. | 25-54 (43.0) | Visit of a registered nurse |
| 2 | Brazil | Sub-study of the Brazilian study of nutrition and health (EBANS) | Scientific article (45)  Methodological article (72) | 2014-2015 | Representative of Brazilian urban population: selected by The participants were selected using a random complex, multistage sampling frame with a random selection of Primary Sampling Unit (PSU) areas (e.g., counties, municipalities, neighborhoods, residential areas) | 575 | 564 | 98.1% | Nonresponse bias was investigated for sex, level of formal education and BMI | Statistical models were adjusted by region, age, race, educational level, marital status, and employment situation. | 18-65 (women:39.7, men: 38.4;) | In person |
| 3 | Canada, wave 1 | Canadian Health Measures Survey (CHMS) | Scientific article (41)  Methodological report (78) | 2007-2009 | Nationally representative sample: selected by stratified random sampling.  The Labour Force Survey (LFS) sampling frame was used to create the collection sites  and control their size. | ±6382^d^ | 3255^d^ | 51%^d^ | No nonresponse bias analysis has been specified. | Weights are used to represent the Canadian population. In accordance with the weighting strategy, the selection weights for collection sites are multiplied  by the selection weights for dwellings (households), adjusted for non-response. Following the  conversion of household weights into person weights, the latter are adjusted for non-response at the  interview stage and the mobile examination centre (MEC) stage, and with several other  adjustments, this weight becomes the final person weight. | 20-79 (NM) | Introductory letter via mail, followed by interviewer visit.  Media launch and local dignitaries and celebrities were invited to see the local clinic and be tested |
|  | Canada, wave 2 | Canadian Health Measures Survey (CHMS) | Scientific article (37)  Methodological report (79) | 2009-2011 | Nationally representative sample: selected by stratified random sampling.  The Labour Force Survey (LFS) sampling frame was used to create the collection sites  and control their size. | 6375^a^ | 2959^b^ | NM | No nonresponse bias analysis has been specified. | The survey weight is calculated as the inverse of the probability that the respondent was selected for the survey. A separate weight was created for the analysis of the activity monitor data, even though all respondents were asked to wear the activity monitor for one week following the MEC appointment. | 18-79  (NM) | Introductory materials via mail, followed by interviewer visit to convince participants |
|  | Canada, wave 3 | Canadian Health Measures Survey (CHMS) | Scientific article (39)  Methodological report (80) | 2012-2013 | Nationally representative sample: stratified random sampling. The sample of dwellings is selected within collection sites in such a way that respondents are able  to travel to the MEC within a reasonable period of time. | 5632^a^ | 2517^b^ | NM | No nonresponse bias analysis has been specified. | The survey weight is calculated as the inverse of the probability that the respondent was selected for the survey. A separate weight was created for the analysis of the activity monitor data, even though all respondents were asked to wear the activity monitor for one week following the MEC appointment. | 18-79 (NM) | Introductory materials via mail, followed by interviewer visit to convince participants |
|  | Canada, wave 4 | Canadian Health Measures Survey (CHMS) | Scientific article (40)  Methodological report (81) | 2014-2015 | Representative sample of the Canadian population: stratified random sampling. The sample of dwellings is selected within collection sites in such a way that respondents are able  to travel to the MEC within a reasonable period of time. | NM | 2390^c^ | NM | No nonresponse bias analysis has been specified. | The survey weight is calculated as the inverse of the probability that the respondent was selected for the survey. A separate weight was created for the analysis of the activity monitor data, even though all respondents were asked to wear the activity monitor for one week following the MEC appointment. | 18-79 (NM) | Introductory materials via mail, followed by interviewer visit to convince participants |
|  | Canada, wave 5 | Canadian Health Measures Survey (CHMS) | Scientific article (39)  Methodological report(82) | 2016-2017 | Nationally representative sample: stratified random sampling. The sample of dwellings is selected within collection sites in such a way that respondents are able  to travel to the MEC within a reasonable period of time. | 5786^a^ | 5657^a^  2355^c^ | NM | No nonresponse bias analysis has been specified. | The survey weight is calculated as the inverse of the probability that the respondent was selected for the survey. A separate weight was created for the analysis of the activity monitor data, even though all respondents were asked to wear the activity monitor for one week following the MEC appointment. | 18-79 (NM) | Introductory materials via mail, followed by interviewer visit to convince participants |
| 4 | England | Sub-study of the Health Survey for England (HSE) | Scientific articles (31,32)  Methodological reports(67,68) | 2008 | Nationally representative sample: randomly selected from the Postcode Address File | 4507 | 2313 | 79.5% | Nonresponse analysis for the whole sample on age and gender. Nothing specified for the PA-subsample | Interview weights, which adjusted for household selection, non-response bias; age, gender and regional profiles were applied in order to produce estimates representing the national population. | 16+ (50.8) | Introductory letter that stated an interviewer would call to set up a home visit. During the home visit participants were asked to wear an accelerometer |
| 5 | Finland, wave 1 | PA and fitness sub-study of the Health 2011 Study | Scientific articles (51,52)  Methodological reports (74) | 2011-2012 | Stratified two-stage cluster sampling design: sample of a previous study and new participants. | 4821 | 2455 | 50.9% | Nonresponse analysis was done for age, sex, education, self-reported work ability, language, self-rated health status and time-use as this is register data. | Register data were used to assess the characteristics of the non-participants  and to construct the survey weights to be used in the analysis. Low participation rate limits generalization of the results to the adult Finnish population. Unequal participation of age- and sex groups limits generalizability of the results. | 18-78 (50.0) | Invitation letter including the date for a scheduled health examination |
|  | Finland, wave 2 | FinFit 2017 | Scientific article (53) | 2017-2019 | Population-based study: stratified random sample from seven regions | 13500 | 2378 | 17.6%^d^ (41% of the sample that was reached by mail or phone) | Nonresponse analysis have been done for multiple register variables (age, gender, language, marital status, area, cardiovascular disease diagnoses, mental health, infections, births and pregnancy, accidents, poisonings and external causes and employment. | The sampling weights were updated to account for differences in the  participation probabilities based on the inverse probability weighting method. | 20-69 (49.5) | Invitation letters with information, afterwards participants were approached by mail or phone |
| 6 | Korea | Sub-study of the Korean National Health and Nutrition Examination Survey (KNHANES) | Scientific article (59)  Methodological article (75) | 2014-2017 | Complex probability-sampling | NM | 2817 | NM | NM | Statistical models were adjusted for sex, age group, educational level, household income, occupational category, alcohol consumption, smoking status, obesity and multimorbidity | ≥20  (51.47) | NM |
| 7 | Latin America ( Argentina, Brazil, Chile, Colombia, Costa Rica, Ecuador, Peru, and Venezuela) | Latin American Study of Nutrition and Health (ELANS) | Scientific articles (43,44)  Methodological report/papers (70,71) | 2014-2015 | Multinational representative sample: selected by complex stratified random sampling | ±3600^d^ | 2737 | 76%^d^ | Nonresponse bias was investigated for sex, age, education and socioeconomic level. | Statistical models were adjusted for sex, age, SEL, and educational level | 15-65 (36.6) | Field interviewer arrived at the home of the participants to explain the study |
| 8 | Luxemboug | Observation of Cardiovascular Risk Factors in Luxembourg 2 (ORISCAV-LUX 2) | Scientific article (42)  Methodological paper (65) | 2016-2018 | Resample of ORISCAV-LUX 1 with 3 additional sampling strategies:   - New random sample - Via the European Health Examination Survey - Volunteers | 1558 | 1213 | 78%^d^ | Nonresponse bias was investigated for sex, age category, district of residence educational level, marital status, country of birth, physical activity, self-reported health perception, cardiometabolic risk profile, blood pressure, BMI | NM | 18-79 (48.4) | Invitation letters sent after which they got contacted by the administrative assistant to fix an appointment |
| 9 | Norway, wave 1 | Kan1 | Scientific article(48) | 2008-2009 | Representative sample: drawn from the Norwegian population registry | 11248 | 3867 | 34% | Statistics Norway completed a dropout analysis that compared factors between those who responded positively and those who were invited but did not respond. The factors analyzed were age, sex, country of birth, number of children, civil status, level of education, and level of income. | Nothing stated about adjustments or weights based on the differences found in the nonresponse analysis | 20-85 (women: 48.3; men: 50.0) | Local media coverage, personalized invitation letters and offer of individual survey report after completion |
|  | Norway, wave 2 | Kan2 | Scientific article(49) | 2014-2015 | Nationally representative sample, but a follow-up sample from the previous wave | 11147 | 5109 (3180 new, 1929 follow-up) participants | 29% in de the new sample, 61% in the follow-up sample | Nonresponse analysis was done for income, education level and for weight. | Nothing stated about adjustments or weights based on the differences found in the nonresponse analysis | 20-85 (women: 44.5; men: 45.6 [20-64 years]; older women: 71.7; older men 71.8 [65+ years]) | Invitation letters and text |
| 10 | Portugal, wave 1 | Portuguese PA and sports monitoring system | Scientific articles (33,58) | 2006-2009 | Representative sample of the Portuguese population: selected by proportionate stratified random sampling taking into account the number of people by age and gender in each region of mainland Portugal | 11746 | 6280 | 53.5% | No nonresponse bias analysis have been reported | No adjustments have been reported | 18+ (NM) | Recruited from schools, work sites and community settings |
|  | Portugal, wave 2 | Portuguese PA and sports monitoring system | Scientific article (55) | 2017-2018 | National representative sample that was selected by proportionate stratified random sampling | NM | 6369 | NM | No nonresponse bias analysis have been reported | No adjustments have been reported | 10+ (NM) | Recruited from schools, work sites and community settings |
| 11 | Singapore | Subsample of the Singapore Health 2 (SH2) study | Scientific article (38)  Methodological paper (66) | 2014-2015 | Nationally representative cross-section survey. Randomly selected through multistage stratified cluster sampling | 2686 | 895 | 33.3%^d^ | Dropout analysis was carried out comparing age group, gender, ethnicity, marital status, educational level, employment status, BMI, smoking status, alcohol drinking, psychological stress, asthma, cancer, diabetes, heart attack, stroke and the presence of at least one disease comparing the accelerometer subsample and those of the overall sample | NM | 18-79 (46.0) | NM |
| 12 | Sweden, wave 1 | ABC | Scientific article(47) | 2001-2002 | Randomly selected from the Swedish population register | 3300 | 1556 | 47.2%^d^ | Dropout analysis (chi-square test) was carried out comparing gender, age groups, and county of residence of the final sample against those of the Swedish population in 2001 and the eligible sample of 2262 | Differences are reported between respondents and non-respondents are mentioned. Nothing said about adjustments that are made | 18-69 (45.0) | A telemarketing company randomly recruited subjects by phone |
|  | Sweden, wave 2 | Follow-up study | Scientific article (46) | 2008 | Same people were approached as in the nationally representative cohort of 2001-2002 | 1114 | 511 | 45.9% | Dropout analysis was carried out comparing gender, age groups, BMI, educational level, perceived health and self-reported exercise of te final sample against those of the sample from the previous study. | No differences in baseline data were found for those who dropped out compared to those who followed the study with regard to sex, BMI, education level, self-reported health, or exercise habits. No adjustments are mentioned. | 18-75 (45.0) | By phone via a telemarketing company |
| 13 | United States, wave 1 | National Health and Nutrition Examination Survey (NHANES) | Scientific article (62)  Methodological reports (69,76) | 2003-2004 | Representative sample of the US civilian noninstitutionalized population. Selected with complex, multistage probability design | 9643 | 7176 | 74.4%^d^ | Nonresponse analyses have been done to compare participants with more and less valid days. | A multistage procedure for nonresponse adjustment was carried out to adjust for unit nonresponse in NHANES for each stage of nonresponse. Adjusted sample weights for subsamples with either one or more or four or more valid days were used for all analyses | 20+ (NM) | Interviewer visits the home of participants during which participants were recruited for the PA study taking place at a mobile examination centre |
|  | United States, wave 2 | National Health and Nutrition Examination Survey (NHANES) | Scientific articles(34,36, 60,63,64)  Methodological reports (69,77) | 2005-2006 | Nationally representative data. Selected with complex, multistage probability design. | 9950 | 4372 | 43.9^d^ | No nonresponse bias analysis have been reported. | A multistage procedure for nonresponse adjustment was carried out to adjust for unit nonresponse in NHANES for each stage of nonresponse. | 20+ (47.1) | Interviewer visits the home of participants during which participants were recruited for the PA study taking place at a mobile examination centre |
|  | United States, wave 3 | National Health and Nutrition Examination Survey (NHANES) | Scientific articles (35, 61) | 2011-2012 | Complex multistage probability sample representative of the US civilian noninstitutionalized population | NM | 6917 | NM | No nonresponse bias analysis have been reported | A multistage procedure for nonresponse adjustment was carried out to adjust for unit nonresponse in NHANES for each stage of nonresponse | 3+ | Interviewer visits the home of participants during which participants were recruited for the PA study taking place at a mobile examination centre |
|  | United States, wave 4 | National Health and Nutrition Examination Survey (NHANES) | Scientific papers (35,61) | 2013-2014 | Complex multistage probability sample representative of the US civilian noninstitutionalized population | NM | 7776 | NM | No nonresponse bias analysis have been reported | A multistage procedure for nonresponse adjustment was carried out to adjust for unit nonresponse in NHANES for each stage of nonresponse | 3+ | Interviewer visits the home of participants during which participants were recruited for the PA study taking place at a mobile examination centre |

Note. NM is not reported in the papers. PA is physical activity. ^a^ this also includes children (age 3+ years). ^b^ this only includes the adults (age 18+ years). ^c^ this only includes participants with enough days with valid data according to the study. ^d^ calculated based on information found in the papers

**Table 2** Study information and sample characteristics of the surveillance systems using pedometers by country and wave

|  | **Country** | **Surveillance system** | **Article(s), type of source** | **Year(s) of monitoring** | **Sample size** | **Number of participants** | **Response rates** | **Age range (mean age) – years** | **Representativeness** | **Recruitment** |
| --- | --- | --- | --- | --- | --- | --- | --- | --- | --- | --- |
| 14 | Czech republic | PA and Inactivity of the Inhabitants of the Czech Republic in the Context of Behavioral Changes | Scientific article (57) | 2008-2013 (annually) | NM | 6509 (over the whole six-year period) | NM | 25-65 (41.4) | Czech national survey: systematic random sampling in a geocoded national address database | In-person face-to-face recruitment at home |
| 15 | Denmark, wave 1 | Sub-study of the Danish National Survey of Diet and PA (DANSDA) | Scientific article (56) | 2007 | 367 | 224 | 61% | 18-75 (45.2) | Nationwide, representative cross-sectional survey. Randomly selected from the Danish civil Registration system | NM |
|  | Denmark, wave 2 | Danish National Survey of Diet and PA (DANSDA) | Scientific article (56) | 2012 | 2924 | 1515 | 52% | 18-75 (47.2) | Nationwide, representative cross-sectional survey. Randomly selected from the Danish civil Registration system | NM |
| 16 | Japan | National Health and nutrition survey of Japan (NHNS-J) | Scientific article (54) | 1995-2007 (annually) | NM | 1995= 9740; 1996 = 9682; 1997= 9404; 1998= 9833; 1999= 8707; 2000= 8473; 2001= 9238; 2002= 8663; 2003= 8367; 2004= 6502; 2005= 6706; 2006= 7124; 2007= 6768 | NM | 20+ (NM) | Randomly selected census units from the whole of Japan | NM |

Note. NM is not reported in the papers

**Table 3** Device information and data collection methods of surveillance systems using accelerometers by country and wave

| **Country and wave** | **Monitor brand and model** | **Anatomical wear side** | **Distribution monitor** | **Epoch length at initialization** | **Wear days** | **Wearing hours** | **Compliance percentage** | **Incentives for participants** |
| --- | --- | --- | --- | --- | --- | --- | --- | --- |
| Barbados | Actiheart | Chest | In person | 15 sec | 7 days | Continuously | 97% | No |
| Brazil | ActiGraph GT3X | Waist /right hip | In person | Sampling rate of 30Hz | 7 days | All waking hours (except during water-based activities) | 92% | No |
| Canada, wave 1 | Actical | Right hip | In person | 60 sec | 7 days | All waking hours | 87% | No |
| Canada, wave 2 | Actical | Right hip | In person | 60 sec | 7 days | All waking hours | 81%* | No |
| Canada, wave 3 | Actical | Right hip | In person | 60 sec | 7 days | All waking hours | NM | No |
| Canada, wave 4 | Actical | Right hip | In person | 60 sec | 7 days | All waking hours | NM | No |
| Canada, wave 5 | Actical | Right hip | In person | 60 sec | 7 days | All waking hours | 73%* | No |
| England | ActiGraph GT1M | Right hip/waist | In person | 60 sec | 7 consecutive days | All waking hours (except during water-based activities) | 59% | £20 gift voucher |
| Finland, wave 1 | Hookie | Right hip | In person | 6 sec | 7 consecutive days | All waking hours (except during water-based activities) | 78%* | Feedback from the measurements |
| Finland, wave 2 | UKKRM42 | Right hip during waking hours, non-dominant wrist when sleeping | In person | 6 sec | 7 consecutive days | All hours except during water-based activities | 95% | Feedback from the measurements |
| Korea | ActiGraph GT3X+ | Left or right hip | In person | 60 sec | 7 consecutive days | During all school- or work-related activities (except during water-based activities) | 80% | NM |
| Latin America | ActiGraph GT3X | Waist | In person | Sampling rate of 30 Hz | 7 consecutive days | All waking hours (except during water-based activities) | 86% | No |
| Luxembourg | ActiGraph GT3X+ | Non-dominant wrist | In person | Sampling rate of 30 Hz | 7 consecutive days | All hours except during water-based activities | 94% | NM |
| Norway, wave 1 | ActiGraph GT1M | Right hip | By mail | 10 sec | 7 consecutive days | All waking hours (except during water-based activities) | 94% | Report on their own PA |
| Norway, wave 2 | ActiGraph  s GT1M and GT3X+ | Right hip | By mail | 60 sec | 7 consecutive days | All waking hours (except during water-based activities) | 96%* | Report on their own PA |
| Portugal | ActiGraph GT1M | Right hip | In person | 15 sec | 4 consecutive days (including two weekend days) | All waking hours (except during water-based activities) | 72.9% - 74.8%^a^ | Report on their own PA |
| Portugal, wave 2 | Actigraph GT3X | Right hip | NM | 15 sec | NM | All waking hours | NM | NM |
| Singapore | Actigraph wGT3X-BT | Right hip | In person | Sampling rate of 30 Hz | 7 consecutive days | All hours or all waking hours (except during water-based activities) | 83% | NM |
| Sweden, wave 1 | ActiGraph 7164 | Lower back | Sent via mail (prepaid envelope) | 60 sec | 7 consecutive days | All waking hours (except during water-based activities) | 78.5%* | The participants were offered a 3-month subscription to a health magazine |
| Sweden, wave 2 | ActiGraph 7164 | Lower back | Sent via mail | 60 sec | 7 consecutive days | All waking hours (except during water-based activities) | 96% | The participants were offered a 3-month subscription to a health magazine |
| United States, wave 1 | ActiGraph 7164 | Right hip | In person | 60 sec | 7 days | All waking hours (except during water-based activities) | 68% | $40 remuneration |
| United States, wave 2 | ActiGraph 7164 | Right hip | In person | 60 sec | 7 consecutive days | All waking hours (except during water-based activities) | 86% | $40 remuneration |
| United States, wave 3 | ActiGraph GT3X+ | Non-dominant wrist | In person | Sampling rate of 80 Hz | 7 consecutive days | 24 hours | 68-80%*^a^ | $40 remuneration |
| United States, wave 4 | ActiGraph GT3X+ | Non-dominant wrist | In person | Sampling rate of 80 Hz | 7 consecutive days | 24 hours | 68-80%*^a^ | $40 remuneration |

Note. NM is not reported. Sec is seconds. * calculated based on information found in the papers. ^a^ papers reported different numbers

**Table 4**  Device information and data collection methods of surveillance systems using pedometers by country and wave

| **Country and wave** | **Monitor brand and model** | **Anatomical wear side** | **Distribution monitor (and return)** | **Wear days** | **Wearing hours** | **Compliance percentage** | **Incentives for participants** |
| --- | --- | --- | --- | --- | --- | --- | --- |
| Czech republic | Yamax Digiwalker SW-700 pedometer | Waist (right-hand side in the midline of the right knee) | NM | 7 consecutive days | All waking hours (except during water-based activities) | 78.7%* | No incentives |
| Denmark, wave 1 | Yamax pedometer | Waist (right-hand side in the midline of the knee) | NM | 7 consecutive days | All waking hours (except during water-based activities) | 91.5%* | NM |
| Denmark, wave 2 | Yamax pedometer | Waist (right-hand side in the midline of the knee) | NM | 7 consecutive days | All waking hours (except during water-based activities) | 93.6%* | NM |
| Japan | Yamax AS 200 pedometer | Waist | NM | Single “typical” day | All waking hours (except during water-based activities) | NM | NM |

Note. NM is not reported. * calculated based on information found in the papers.

**Table 5** Data processing and outcome measures of the surveillance system using accelerometer by country and wave

| **Country and wave** | **Epoch length during analysis** | **Minimum number of valid days need** | **Non-wear time definition** | **Wear time definition for valid day** | **PA intensity cut-points (cpm; steps/day)** | **Treatment of outliers** | **Outcome measures reported in the identified papers** |
| --- | --- | --- | --- | --- | --- | --- | --- |
| Barbados | 15 sec | 48-72 hours of cumulative wear | Windows of an hour or more wherein the device was inferred to be completely stationary, where stationary is defined as standard deviation in each axis not exceeding the approximate baseline noise of the device itself | 24 hours | MET (95):  SB < 1.5  LPA 1.5-3  MVPA ≥3 | NA | PA energy expenditure;  Min/d SB, LPA, MVPA;  PA inactivity (95) |
| Brazil | 60 sec | ≥ 5 days (at least one weekend day) | Any periods of continuous zero counts for ≥60 consecutive min | ≥10 hr | Cpm (96):  SB ≤100  Mild PA = 101-1951  MPA =1952-5724  VPA ≥5725  MVPA ≥1952 | Normal analyses | Min/d SB, LPA, MPA, MVPA;  Steps/d;  Adherence to PA guidelines (95) |
| Canada, wave 1 | 60 sec | ≥4 days (at least one weekend day) | Any periods of continuous zero counts for ≥60 consecutive min (with allowance for up to 2 min of up to 100 counts interruptions) | ≥10 hr | Cpm (97):  SB < 100  LPA = 100-1535  MPA 1535-3962  VPA ≥ 3962  MVPA ≥ 1535 | spurious data points were anything >20,000cpm. If >15 spurious data points were present for a given respondent, the data was not used. If fewer than 15 spurious data points were present, they were replaced with an imputed value of the average of the two surrounding data points. | Adherence to PA guidelines (98); Min/d of SB, LPA, MPA, VPA, MVPA;  Steps/d;  Time in sedentary bouts ≥20 min; number of sedentary breaks |
| Canada, wave 2 | 60 sec | ≥4 days (at least one weekend day) | Any periods of continuous zero counts for ≥60 consecutive min (with allowance for up to 2 min of up to 100 counts interruptions) | ≥10 hr | Cpm (97):  SB < 100  MVPA ≥ 1535 | spurious data points were anything >20,000cpm. If >15 spurious data points were present for a given respondent, the data was not used. If fewer than 15 spurious data points were present, they were replaced with an imputed value of the average of the two surrounding data points. | Adherence to PA guidelines (98);  Min/d of SB, MVPA, time in sedentary bouts ≥20 min;  Number of sedentary breaks |
| Canada, wave 3 | 60 sec | ≥4 days (at least one weekend day) | Any periods of continuous zero counts for ≥60 consecutive min (with allowance for up to 2 min of up to 100 counts interruptions) | ≥10 hr | Cpm (97):  SB < 100;  MPA 1535-3961  VPA ≥ 3962 | spurious data points were anything >20,000cpm. If >15 spurious data points were present for a given respondent, the data was not used. If fewer than 15 spurious data points were present, they were replaced with an imputed value of the average of the two surrounding data points. | Adherence to PA guidelines (98);  Min/d of MVPA, MPA, VPA |
| Canada, wave 4 | 60 sec | ≥4 days (at least one weekend day) | Any periods of continuous zero counts for ≥60 consecutive min (with allowance for up to 2 min of up to 100 counts interruptions) | ≥10 hr | Cpm (97):  SB < 100  MVPA ≥ 1535 | spurious data points were anything >20,000cpm. If >15 spurious data points were present for a given respondent, the data was not used. If fewer than 15 spurious data points were present, they were replaced with an imputed value of the average of the two surrounding data points.  Excluded if more than 6 standard deviations above the mean | Adherence to PA guidelines (98);  Min/d of MVPA, MPA, VPA, bouts of MVPA |
| Canada, wave 5 | 60 sec | ≥4 days (at least one weekend day) | Any periods of continuous zero counts for ≥60 consecutive min (with allowance for up to 2 min of up to 100 counts interruptions) | ≥10 hr | Cpm (97):  SB < 100  MVPA ≥ 1535 | spurious data points were anything >20,000cpm. If >15 spurious data points were present for a given respondent, the data was not used. If fewer than 15 spurious data points were present, they were replaced with an imputed value of the average of the two surrounding data points. | Adherence to PA guidelines (98);  Min/d of MVPA, MPA, VPA |
| England | 60 sec | ≥4 days | any periods of continuous zero counts for ≥60 consecutive min | ≥10 hr | Cpm (62):  SB =0-199  LPA =200-2019  MPA = 2020-5998  VPA ≥ 5998  MVPA ≥ 2020 | NM | Average number of minutes/d of SB, LPA, MPA and VPA;  Physically active (≥150 min of MVPA/wk);  Physically inactive (<150 min MVPA/wk) |
| Finland, wave 1 | 6 sec | ≥4 days | If raw peak-to-peak acceleration remained <562.5 mg for each axis at least 30 minutes. | ≥10 hr | METs (99):  LPA = 1.5-2.9  MPA = 3.0-5.9  VPA ≥6.0  From epoch-wise mean amplitude deviation to MET (100) | Participants whose daily measurement time was over 20h were considered to have slept with the accelerometer. Their waking time was limited to 20h and the exceeding time was reduced from the lying time. | Min/wk MPVA; meeting the PA guidelines (99);  hours/day SB, MVPA;  number of breaks in SB |
| Finland, wave 2 | 6 sec | ≥4 days | If continuous quiescent time is longer than 120 min | 24 hr | METs (100):  SB < 1.5  LPA = 1.5 - 3.0  MVPA ≥3.0 | Acceleration higher than human can reach (e.g. accelerometer is in washing machine), those days are excluded. | Min/d SB, standing, LPA & MVPA;  Steps/d;  Steps/hr;  Min MVPA/hr |
| Korea | NM | ≥4 days | any periods of continuous zero counts for ≥60 consecutive min | ≥10 hr | CPM (101):  SB ≤100;  LPA = 100-759  LA = 760-2019  MVPA ≥ 2020 | NM | Min/d SB, LPA, LA, MVPA, adherence to PA and/or SB guidelines |
| Latin America | NM | ≥ 5 (at least one weekend day) | any periods of continuous zero counts for ≥60 consecutive min | ≥10 hr | Cpm (96):  SB ≤100;  MPA =1952-5724; VPA≥5725;  MVPA ≥1952 | Normal analyses | Min/d SB, MPA, VPA, MVPA |
| Luxembourg | 5 sec | ≥ 4 (at least one weekend day) | NM | ≥10 hr | Acceleration intensity thresholds (mg) (102, 103):  SB<44.8  MVPA>428 | NM | Min/day SB, LPA, MVPA  Adherence to guidelines |
| Norway, wave 1 | 60 sec | ≥4 days | any periods of continuous zero counts for ≥60 consecutive min (with allowance for up to 1 min of > 0 cpm) | ≥10 hr | Cpm:  SB < 100 (104)  LPA = 100-759  LA = 760-2019 (105, 106)  MVPA ≥ 2020 (62) | NM | Steps/d;  Min/d SB, LPA, LA, MVPA;  Adherence to PA guidelines;  ≥10,000 steps/d |
| Norway, wave 2 | NM | ≥ 1 day | any periods of continuous zero counts for ≥60 consecutive min (with allowance for up to 2 min of > 0 cpm) | ≥10 hr | Cpm (62):  SB < 100,  LPA = 100-2019  MVPA ≥2020  VPA = NM | NM | Min/d SB, LPA, MVPA, VPA; MVPA in 10-min bouts;  adherence to PA guidelines |
| Portugal | 60 sec | ≥3 days(at least one weekend day) | any periods of continuous zero counts for ≥60 consecutive min | ≥10 hr | Cpm (62):  SB <100;  LPA = 100-2019; MVPA ≥2020;  MPA = 2020-5998  VPA ≥ 5999 | Records of PA above 1500 counts/min were excluded | Min/d SB, LPA, MVPA  Breaks in sedentary time;  Number and time in sedentary bouts/d;  10-min MVPA bouts |
| Portugal, wave 2 | 60 sec | ≥3 days(at least one weekend day) | any periods of continuous zero counts for ≥60 consecutive min | ≥10 hr | Cpm (62):  SB>100  MPVA ≥2020 | NM | Min/day SB, LPA, MPA, VPA, breaks in sedentary time, number of breaks/SB hour |
| Singapore | 60 sec | ≥4 days | 90-min consecutive zero counts (with allowance for up 2 min interval interruption) | ≥10 hr | Cpm:  SB<150 (107)  MVPA ≥ 2690 (108) | NM | Granular spectrum of activity intensities, 1-, 2-, 5-, 10, 15-min bout MVPA |
| Sweden, wave 1 | NM | ≥4 days (at least one weekend day) | Any periods of continuous zero counts for ≥20 minutes | ≥10 hr | Cpm (96):  SB < 100  MPA = 1952-5724 VPA ≥5725 | Files with counts/min > 20000 were excluded. | 2-min & 10-min bouts of MVPA;  Min/d SB, MPA, VPA, MVPA |
| Sweden, wave 2 | NM | ≥4 days | any periods of continuous zero counts for ≥60 consecutive min (with allowance for up to 2 min of up to 100 counts) | ≥10 hr | Cpm:  SB < 100 (104)  LPA =100-2019 (62)  MVPA ≥ 2020 | 5 accelerometer were malfunctioning, no extreme outliers was observed. | Min/d SB, LPA, MVPA |
| United States, wave 1 | 60 sec | ≥4 days | any periods of continuous zero counts for ≥60 consecutive min (with allowance for up to 2 min of up to 100 counts) | ≥10 hr | Cpm (96, 109-111):  MPA = 2020-5998  VPA ≥ 5999 | No formal outlier detection was conducted | Cpm during wear time;  Min/d MPA, VPA and MVPA;  Adherence to PA guidelines (112) |
| United States, wave 2 | 60 sec | ≥ 1 day | daily time worn (hours and minutes) was computed by using a SAS marco provided by the National Cancer Institute (NCI) | ≥10 hr | Cpm:  LA =760-2019 (112)  Inactive = 0-499 (113)  LPA = 500-2019  MPA= 2020-5998  VPA ≥5999; | Records containing >10min with 1) zero steps and >250 activity counts per minute, 2) >200 steps per minute, and 3) 32,767 (maximum value possible) activity counts per minute were red flagged and excluded | Min/d at LA, LPA, MPA, VPA and MVPA;  Adherence to PA guidelines |
| United States, wave 3 | 90 sec | ≥4 days (at least one weekend day) | Non-wear was calculated by a machine learning algorithm using a three-step process | ≥1380 valid min, <72 min of non-wear time and <17h of sleep | A higher Monitor-Independent Movement Summary (MIMS) value indicates a higher level of overall PA. | NM | MIMS value |
| United States, wave 4 | 90 sec | ≥4 days (at least one weekend day) | Non-wear was calculated by a machine learning algorithm using a three-step process | ≥1380 valid min, <72 min of non-wear time and <17h of sleep | A higher MIMS value indicates a higher level of overall PA. | NM | MIMS value |

Note. NM is not reported. NA is not applicable. Cpm is counts per minute. Hr is hour. SB is sedentary behaviour, LPA is low physical activity, MPA is moderate physical activity, VPA is vigorous physical activity, MPVA is moderate-to-vigorous intensity physical activity, LA is lifestyle activity.

**Table 6** Data processing and outcome measures of the surveillance system using pedometers by country and wave

| **Country and wave** | **Minimum number of valid days need** | **Non-wear time definition** | **Wear time definition for valid day** | **PA intensity cut-points (cpm; steps/day)** | **Treatment of outliers** | **Outcome measures reported in the identified papers** |
| --- | --- | --- | --- | --- | --- | --- |
| Czech Republic | 7 days | *Non-wear time was recorded in a self-administered step diary* | ≥10 hr | inactive lifestyle: <7500 steps/day  very active lifestyle: > 12.500 steps/day  (“zone” approach (114)) | the steps/day values lower than 1000 and higher than 25000 were considered as outlier | Steps/d;  PA lifestyle; |
| Denmark, waves 1 and 2 | ≥4 days | *Non-wear time was recorded in a self-administered step diary* | ≥10 hr | Steps/day (115):  SB < 5000  low active = 5000-7499  somewhat active = 7500-9999  active = 10000-12499  highly active ≥12500. | Steps/d <100 or >50.000 were treated as outliers | Steps/d;  Step-defined activity level |
| Japan | 1 day | NM | NM | NM | NM | Steps/d |

Note. NM is not reported. Hr is hour. D is day. PA lifestyle is physical active lifestyle.

Additional references

95. Global Recommendations on Physical Activity for Health. Geneva: World Health Organization 2010.

96. Freedson PS, Melanson E, Sirard J. Calibration of the Computer Science and Applications, Inc. accelerometer. Medicine & Science in Sports & Exercise. 1998 May;30(5):777–81.

97. Colley RC, Tremblay MS. Moderate and vigorous physical activity intensity cut-points for the Actical accelerometer. Journal of Sports Sciences. 2011 May;29(8):783–9.

98. Warburton DE, Charlesworth S, Ivey A, Nettlefold L, Bredin SS. A systematic review of the vidence for Canada’s Physical Activity Guidelines for Adults. International Journal of Behavioral Nutrition and Physical Activity. 2010;7(1):39.

99. 2008 physical activity guidelines for Americans: be active, healthy and happy! Rockville: Department of Health and Human Services; 2008.

100.Vähä-Ypyä H, Vasankari T, Husu P, Mänttäri A, Vuorimaa T, Suni J, et al. Validation of Cut-Points for Evaluating the Intensity of Physical Activity with Accelerometry-Based Mean Amplitude Deviation (MAD). Miller PJO, editor. PLOS ONE. 2015 Aug 20;10(8):e0134813.

101. Kim J, Tanabe K, Yokoyama N, Zempo H, Kuno S. Objectively measured light-intensity lifestyle activity and sedentary time are independently associated with metabolic syndrome: a cross-sectional study of Japanese adults. International Journal of Behavioral Nutrition and Physical Activity. 2013;10(1):30.

102. Hildebrand M, Van Hees VT, Hansen BH, Ekelund U. Age Group Comparability of Raw Accelerometer Output from Wrist- and Hip-Worn monitors.Med Sci Sport Exerc. 2014;46:1816–24.

103. Hildebrand M, Hansen BH, van Hees VT, Ekelund U. Evaluation of raw acceleration sedentary thresholds in children and adults. Scand J Med Sci Sports. 2017;27:1814–23.

104. Matthews CE, Chen KY, Freedson PS, Buchowski MS, Beech BM, Pate RR, et al. Amount of Time Spent in Sedentary Behaviors in the United States, 2003-2004. American Journal of Epidemiology. 2008 Mar 14;167(7):875–81.

105.Hagstromer M, Troiano RP, Sjostrom M, Berrigan D. Levels and Patterns of Objectively Assessed Physical Activity--A Comparison Between Sweden and the United States. American Journal of Epidemiology. 2010 Apr 20;171(10):1055–64.

106. MAatthews CE. Calibration of Accelerometer Output for Adults. Medicine & Science in Sports & Exercise. 2005 Nov;37(Supplement):S512–22.

107. Kozey-KeadleE S, Libertine A, Lyden K, Staudenmayer J, Freedson PS. Validation of Wearable Monitors for Assessing Sedentary Behavior. Medicine & Science in Sports & Exercise. 2011 Aug;43(8):1561–7.

108. Sasaki JE, John D, Freedson PS. Validation and comparison of ActiGraph activity monitors. Journal of Science and Medicine in Sport. 2011 Sep;14(5):411–6.

109. Brage S, Wedderkopp N, Franks PW, Bo Andersen L, Froberg K. Reexamination of Validity and Reliability of the CSA Monitor in Walking and Running. Medicine & Science in Sports & Exercise [Internet]. 2003;35(8):1447–54.

110. Leenders NY, Sherman WM, Nagaraja HN, Kien CL. Evaluation of methods to assess physical

activity in free-living conditions. Med Sci Sports Exerc. 2001;33(7):1233-40

111. Yngve A, Nilsson A, Sjostrom M, Ekelund U. Effect of Monitor Placement and of Activity Setting on the MTI Accelerometer Output. Medicine & Science in Sports & Exercise. 2003 Feb;35(2):320–6.

112.USDHHS. Physical activity and health: a report of the Surgeon General Atlanta, GA: US. U.S. Department of Health and Human Services, Centers for Disease Control and Prevention, National Center for Chronic Disease Prevention and Health Promotion; 1996.

113. Tudor-Locke C, Ainsworth BE, Thompson RW, Matthews CE. Comparison of pedometer and accelerometer measures of free-living physical activity. Med Sci Sports Exerc. 2002;34(12):2045–51.

114. Tudor-Locke C, Hatano Y, Pangrazi RP, Kang M. Revisiting “How Many Steps Are Enough?” Medicine & Science in Sports & Exercise. 2008 Jul;40(Supplement):S537–43.

115.Tudor-Locke C, Bassett DR. How Many Steps/Day Are Enough? Sports Medicine 2004;34(1):1–8.
